# A Rare Case of Glycogen Storage Disease Type 1a Presenting with Hemophagocytic Lymphohistiocytosis (HLH)

**DOI:** 10.1155/2020/8818617

**Published:** 2020-11-11

**Authors:** Hedyeh Saneifard, Bibishahin Shamsian, Marjan Shakiba, Simin Karizi Zarea, Ali Sheikhy

**Affiliations:** ^1^Mofid Children Hospital, Shahid Beheshti University of Medical Sciences, Tehran, Iran; ^2^Pediatric Congenital Hematologic Disorders Research Center, Shahid Beheshti University of Medical Sciences, Tehran, Iran; ^3^Tehran University of Medical Sciences, Tehran, Iran

## Abstract

**Background:**

Hemophagocytic lymphohistiocytosis (HLH) is a life-threatening hyperinflammatory syndrome characterized by fever, respiratory distress, massive hepatomegaly, and bicytopenia. It is classified into primary (congenital) and secondary (acquired) types. There are many diseases associated with secondary HLH, but glycogen storage disease is a novel cause of secondary HLH. *Case Presentation*. In this case, we present a five-month-old female infant with recurrent fever, poor feeding, pallor, and prolonged diarrhea for two months. With a diagnosis of HLH, the patient was treated with IVIG and prednisolone. After treatment was initiated, the patient's general condition improved. All metabolic workup was normal. We did whole-exome sequencing that confirmed glycogen storage disease (GSD) type 1.

**Conclusion:**

Metabolic diseases are one of the severe causes of secondary HLH in infants; hence, complete metabolic assessment is mandatory in these patients, and GSD must be included in the differential diagnosis of HLH metabolic causes.

## 1. Introduction

Hemophagocytic lymphohistiocytosis (HLH) is an inappropriate activation and proliferation of lymphohistocytes. Macrophages and T cells are the primary pathogens in this condition. Altered function of natural killer (NK) cells and a cytotoxic T lymphocyte (CTL) following dysregulated response to antigen-presenting cells (APCs) is the known mechanism for this condition. HLH diagnosis is based on eight criteria (fever, splenomegaly, cytopenia in 2 of 3 lineages, elevated triglycerides or decreased fibrinogen, hemophagocytosis, low or absent NK-cell activity, high ferritin, and elevated soluble CD25), and diagnosis establish with existence of 5 out of 8 criteria. There are two major classifications for HLH, primary, and secondary type. Primary HLH, which is a fatal autosomal recessive disease, begins during infancy or early childhood. A positive familial history following a clinical picture with the absence of a known malignant, rheumatologic, and metabolic disease suggests primary HLH [1]. Secondary HLH associates with infections, malignancies, and rheumatologic and metabolic disorders [2]. Childhood metabolic diseases such as lysinuric protein intolerance, methylmalonic acidemia, propionic acidemia (PA), galactosemia, Gaucher disease, multiple sulfatase deficiencies, Pearson syndrome, galactosialidosis, and biotinidase deficiency were noted to be associated with HLH [3]. Both metabolic evaluation and gene mutation analysis performed simultaneously, especially in infants presenting with HLH, are essential for early diagnosis of metabolic diseases [4]. We present a case of secondary HLH following glycogen storage disease (GSD) type 1, which is extremely a rare etiology of secondary HLH.

## 2. Case Presentation

A 5-month-old female infant was admitted with recurrent fever, poor feeding, pallor, and prolonged diarrhea for two months. She was the second child of consanguineous parents. The pregnancy was uneventful, term, and the child's birth weight was 3100g. In her past medical history, urinary tract infection and vesicoureteral reflux were noted. In physical examination, she was ill with respiratory distress, respiratory rate = 46/min, pulse rate = 150/min, axillary temperature = 38.5°C, blood pressure = 85/60 mmHg, weight = 5 kg (3^rd^ centile), height = 60 cm (10^th^ centile), and head circumference = 40 cm (10^th^ percentile). Pallor, severe hepatosplenomegaly, hypotonia, and seizure were the other findings. CBC, VBG, and blood chemistry of the patient is noted in Table 1.

In abdominal sonography, the liver span was 126 mm (reference range: 60–100 mm) with increased echogenicity, spleen size reported to be 63 mm (reference range: up to 65 mm), and mild fullness in the left kidney with suspicion of nephrocalcinosis was noticed. Chest X-ray revealed mild cardiomegaly. ECG was low voltage with no paroxysmal discharge. Echocardiography reported mild LVH and diastolic dysfunction. She admitted to intensive care unit due to critical condition, persistent metabolic acidosis, which did not respond to intravenous bicarbonate therapy, and decreased level of consciousness. After medical therapy with antibiotics, HLH was suspected due to five positive criteria (Table 2) including fever which persisted for two weeks, splenomegaly, bicytopenia (leukopenia and anemia), hypertriglyceridemia (TG = 2316 mg/dl), low fibrinogen level (<1.5 mg/dL), increased ferritin (>500 ng/ml), and supportive criteria (hypoalbuminemia, neurologic symptoms, and abnormal LFT).

After hematologic consultation, bone marrow aspiration was done, there were no phagocytic cells, but according to fulfilling of HLH criteria, diagnosis of HLH was made. We started 1 g/kg IVIG for two days and 10 mg/m^2^ dexamethasone for ten days that tapered and discontinued in two weeks. The patient responded dramatically to treatments, fever stopped, hemoglobin increased to 11 mg/dl and WBC increased to 7300/*μ*L, ESR dropped, and the patient's general condition improved.

With suspicion to secondary HLH following metabolic disorders, metabolic workup was done.

The results were as follows: ammonia = 257 mmol/L, lactate = 110 mg/dl, pyruvate = 1.24 mg/dl, plasma amino acid chromatography HPLC and homocysteine = normal, urine amino acid chromatography = normal, urine sugar chromatography = +2 glucose band, acylcarnitine and urine organic acid profile = normal.

For diagnosing the cause of secondary HLH, whole-exome sequencing (WES) was done, and the result was homozygous *G6PC* variant that was disease-associated and diagnosis of GSD type 1a was confirmed (Table 3).

Intravenous glucose and bicarbonate was prescribed in hospital setting, and she discharged with prescription of uncooked cornstarch and Shohl's solution. Also, frequent feeding was recommended to prevent hypoglycemia.

## 3. Discussion

Hemophagocytic lymphohistiocytosis most frequently affects infants from birth to 18 months of age, but the disease may also be observed in children and adults of all ages. The pathophysiology of HLH is multifactorial [3].

There are two major types of this condition. Primary HLH caused by gene mutation includes *FHL1, FHL2*(PRF1/perforin), *FHL3* (UNC13D/Munc13-4), *FHL4* (STX11/syntaxin 11), *FHL5* (STXBP2/Munc18-2), *GS2* (RAB27A), *HPS2, XLP1, XLP2, BLOC1S6,CD27, ITK, LYST, MAGT1* (XMEN), *SLC7A7*, and *XIAP*(BIRC4), which they are autosomal recessively transmitted [5].

Secondary HLH may develop secondary to an infection, malignancies, and rheumatologic and metabolic disorders. The most common infection associated with acquired HLH is Epstein–Barr virus (EBV). Malignancy-associated hemophagocytic syndrome (MAHS) is HLH that develops in the presence of malignancy. This condition may cause by autoimmune disorders such as systemic lupus erythematosus, adult-onset Still's disease, and rheumatoid arthritis. Childhood metabolic diseases such as lysinuric protein intolerance, methylmalonic acidemia, propionic acidemia (PA), galactosemia, Gaucher disease, multiple sulfatase deficiencies, Pearson syndrome, galactosialidosis, and biotinidase deficiency were noted to be associated with HLH. Metabolic disease screening tests and gene mutation analysis are crucial, especially when HLH presents in infancy.

Diagnosis is made by existence five out of eight diagnostic criteria. These criteria are as follows: (1) fever ≥38.3°C, (2) splenomegaly, (3) cytopenia (affecting at least two of three lineages in the peripheral blood): hemoglobin <9 g/dL (in infants <4 weeks: hemoglobin <10 g/dL), platelets <100 × 10^3^/mL, and neutrophils <1 × 10^3^/mL), (4) hypertriglyceridemia (>265 mg/dL) and/or hypofibrinogenemia (<150 mg/dL), (5) hemophagocytosis in bone marrow or spleen or lymph nodes or liver, (6) low or absent NK-cell activity, (7) ferritin >500 ng/mL, and (8) elevated soluble CD25 (soluble IL-2 receptor alpha) [6].

Aggressive treatment is needed immediately after diagnosis. The standard of care for HLH is treatment with etoposide and dexamethasone [7]. Intravenous immunoglobulins (IVIg) have been reported as giving good results in infectious, but also autoimmune-related forms of hemophagocytic lymphohistiocytosis (HLH), but only in case reports and small retrospective studies [8].

GSD type Ia or Von Gierke disease or glucose-6-phosphatase (G6Pase) deficiency is caused by a lack of hydrolase subunit of G6Pase. This impairment makes G6Pase nonfunctional. Following this condition, free glucose is not available in the last step of gluconeogenesis, leading to impaired glucose hemostasis and hypoglycemia [9]. The diagnosis is based on the clinical presentation, a specific constellation of biochemical abnormalities (including hyperlipidemia, hyperuricemia, and hypoglycemia), molecular genetic testing, or enzyme activity in liver biopsy tissue. Our patient fulfilled five out of eight HLH criteria, so we initiated HLH treatment. Due to the high suspicion of metabolic disease and following normal metabolic workup, we preformed WES to diagnose the cause of secondary HLH. Detecting a homozygous mutation p.(Arg83Cys) c.247C > T in the *G6PC* in chromosome 17 confirmed GSD type 1a in our patient [10].

The reason that HLH occurs following metabolic diseases is not recognized. However, it may be associated with hyperinflammation condition that leads to inciting factors of HLH, after NK cells, lymphocytes, and macrophages become increasingly activated, and they secrete high levels of cytokines and chemokines. HLH associated with GSD is a rare condition, and it has been only reported twice (Table 4).

The first one was reported by Yeter Düzenli Kar et al. They reported an infant with a history of increased respiratory rate, distended abdomen, and fever for three days. Bone marrow aspiration was performed due to the presence of fever, cytopenia, and hepatomegaly. Hemophagocytosis was observed in the bone marrow by Wright staining. Like in our case, metabolic workup was normal, and *G6PC* gene analysis was performed according to the positive familial history of GSD type 1 in the patient. The final GSD type 1 diagnosis was made by detecting a p.Arg83Cys (c.247C > T) mutation in the *G6PC* gene [11].

The second case by Ang Wei et al. reported an 11-month-old male infant with recurrent fever and icteric sclera for two months. During further investigation, they diagnosed HLH based on fever, bicytopenia, hypofibrinogenemia, decreased NK-cell activity, and hemophagocytosis in a bone marrow aspirate. For evaluation of the cause, gene analysis was done, and they founded c.1544G > A (p.R515H) mutation, which is known cause of type IV GSD [12].

## 4. Conclusion

In the evaluation of a patient with HLH, secondary causes must be considered by the physician. Metabolic diseases are among the severe causes of secondary HLH in infants; hence complete metabolic assessment is mandatory in these patients. Besides other known metabolic causes of HLH, GSD must be included in the differential diagnosis.

## Figures and Tables

**Table 1:** Laboratory findings of the patient.

CBC		Blood chemistry	

WBC	2300/*μ*L (PMN = 48% lymph = 52%)	BS	55 mg/dl
Hb	7.9 mg/dl	AST	1280 *μ*/l
RBC	2.5 × 10^6^/*μ*L	ALT	571 *μ*/l
MCV	100 fl	Total protein	4.3 g/dl
Plt	564 × 10^3^/*μ*L	Alb	2.5 g/dl
ESR	110 mm/h	Chol	1402 mg/dl
CRP	1 mg/ml	TG	2316 mg/dl
Retic	1.7%	Uric acid	3.3 mg/dl
Coombs	Neg	PT	>30 sec
LDH	931 u/L	INR	6

VBG		PTT	>120 sec

pH	7.28	Amylase	22 *μ*/l
p CO_2_	24 mmHg	Lipase	11 *μ*/l
HCO_3_	10 mmol/L	Ferritin	>800 ng/ml
pO_2_	85 mmHg	D-dimer	>200 *μ*/ml

WBC, white blood count (reference range: 6000–17500/*μ*L); Hb, hemoglobin (reference range: 9.5–14.1 mg/dl); RBC, red blood count (reference range: 2.7–4.5 × 10^6^/*μ*L); MCV, mean corpuscular volume (reference range: 72–82 fl); Plt, platelet (reference range: 150–450 × 10^3^/*μ*L); ESR, erythrocyte sedimentation rate (reference range:0–20 mm/h); CRP, C-reactive protein (reference range: <2 mg/ml in this medical center); retic (reference range: 0.5–1.5%); LDH, lactate dehydrogenase (reference range: 500–920 *μ*/l); pH (reference range: 7.34–7.46); pCO_2_ (reference range: 26–41 mmHg); HCO_3_ (reference range:20–24 mmol/l); pO_2_ (reference range: 25–40 mmHg); BS, blood sugar (reference range: 70–110 mg/dl); AST, aspartate aminotransferase (reference range:20–60 *μ*/l); ALT, alanine transferase (reference range: 6–50 *μ*/l); total protein (reference range: 5.6–7.2 g/dl); Alb, albumin (reference range: 3.9–5.1 g/dl); cholesterol (reference range: 50–120 mg/dl); TG, triglyceride (reference range: 20–150 mg/dl); uric acid (reference range:2–6.2 mg/dl); PT, prothrombin time (reference range: 12.2–15.5 sec); INR, international normalized ratio (reference range: <1); PTT, partial thromboplastin time (reference range:26.5–35.5 sec); amylase (reference range: 30–115 *μ*/l); lipase (reference range: 25–120 *μ*/l); ferritin (reference range: 36–391 ng/ml); D-dimer (reference range: <3.4 *μ*/ml).

**Table 2:** HLH diagnostic criteria.

Our patient	HLH criteria	
Axillary temperature = 38.5°C	Fever (defined as a temperature >100.3 F, >38°C)	✓
Splenomegaly	Enlargement of the spleen	✓
Bicytopenia: Hb = 7.9 mg/dl; WBC = 2300/*μ*l	Decreased blood cell counts affecting at least two of three lineages in the peripheral blood	✓
Hypertriglyceridemia: 2316 mg/dl	High blood levels of triglycerides (fasting, greater than or equal to 265 mg/100 ml) and/or decreased amounts of fibrinogen in the blood (≤150 mg/100 ml)	✓
Increased ferritin: >800 ng/ml	Ferritin ≥500 ng/ml	✓
None	Hemophagocytes in the bone marrow, spleen, or lymph nodes	✘
Not checked	Low or absent natural killer cells' activity	✘
Not checked	Soluble CD25 (soluble IL-2 receptor) > 2400 U/ml (or per local reference laboratory)	✘

**Table 3:** WES report.

Gene	Variant coordinates	Zygosity	In silico parameters	Allele frequencies	Type and classification
*G6PC*	Chr17(GRCh37):g.41055964C > T NM_000151.3:c.247C > T p.(Arg83Cys) Exon 2	Homozygous	PolyPhen: probably damaging Align-GVGD: C0 SIFT: deleterious MutationTaster: disease causing Conservation: nt moderate/aa high	gnomAD: 0.00055 ESP: 0.00046 1000 G: CentoMD: 0.0012	Missense pathogenic (class 1)

**Table 4:** Comparison of patient characteristics from previous reported cases.

	Saneifard et al. (2020)	Düzenli Kar et al. (2018)	Wei et al. (2019)
Presenting symptoms	Increased respiratory rate, hepatomegaly, recurrent fever, poor feeding, prolonged diarrhea	Increased respiratory rate, hepatomegaly, fever, decreased blood pressure	Recurrent fever, icteric sclera, hepatosplenomegaly
Consanguinity of parents	Consanguineous	Consanguineous	Not reported
Patient's age	5 months	5 months	11 months
Duration of symptoms	2 months	3 days	2 months
Liver function tests	Elevated	Elevated	Elevated
TG level	Elevated	Elevated	Not reported
Hypoglycemia	Positive	Positive	Not reported
Bicytopenia	Positive	Positive	Positive
Fibrinogen level	Decreased	Decreased	Decreased
Ferritin level	Elevated	Elevated	Elevated
LDH level	Elevated	Elevated	Elevated
Metabolic acidosis	Yes	Yes	Not reported
Genetic study	p.(Arg83Cys) (c.247C > T) mutation in the G6PC gene (homozygote)	p.(Arg83Cys) (c.247C > T) mutation in the G6PC gene (homozygote)	p.(Asp941Asn) (c.2821G > A) mutation in the UNC13D gene (heterozygous)

## Data Availability

All essential data are included within the manuscript.
